# Composition of *Lycium barbarum* polysaccharides and their apoptosis-inducing effect on human hepatoma SMMC-7721 cells

**DOI:** 10.3402/fnr.v59.28696

**Published:** 2015-11-11

**Authors:** Qian Zhang, Xiaoling Lv, Tao Wu, Qian Ma, Anguo Teng, Ying Zhang, Min Zhang

**Affiliations:** 1 Key Laboratory of Food Nutrition and Safety (Tianjin University of Science and Technology), Ministry of Education, Tianjin, China; 2 Department of Biological and Food Engineering, Tianshi College, Tianjin, China

**Keywords:** *Lycium barbarum* polysaccharide, structure, apoptosis, cell cycle, [Ca^2+^]_i_, hepatoma SMMC-7721 cells

## Abstract

**Background:**

*Lycium barbarum* polysaccharide (LBP) is a natural functional component that has a variety of biological activities. The molecular structures and apoptosis-inducing activities on human hepatoma SMMC-7721 cells of two LBP fractions, LBP-d and LBP-e, were investigated.

**Results:**

The results showed that LBP-d and LBP-e both consist of protein, uronic acid, and neutral sugars in different proportions. The structure of LBP was characterized by gas chromatography, periodate oxidation, and Smith degradation. LBP-d was composed of eight kinds of monosaccharides (fucose, ribose, rhamnose, arabinose, xylose, mannose, galactose, and glucose), while LBP-e was composed of six kinds of monosaccharides (fucose, rhamnose, arabinose, mannose, galactose, and glucose). LBP-d and LBP-e blocked SMMC-7721 cells at the G0/G1 and S phases with an inhibition ratio of 26.70 and 45.13%, respectively, and enhanced the concentration of Ca^2+^ in the cytoplasm of SMMC-7721.

**Conclusion:**

The contents of protein, uronic acid, and galactose in LBP-e were much higher than those in LBP-d, which might responsible for their different bioactivities. The results showed that LBP can be provided as a potential chemotherapeutic agent drug to treat cancer.

Liver cancer rates are continuously increasing in Asia because of the persistently high incidence of hepatic disease (1). Many researches have focused on searching for anticancer drugs with higher activities and lower toxicity from nature (2). Traditional Chinese herbs, with anticancer properties, have drawn a great deal of attention in recent years. Investigations have revealed that the anticancer activities of these herbs are partially owing to their capacity to induce apoptosis in cancer cells (3). Apoptosis is different from cell death; it is an initiative process controlled by the genes, so as to adapt better to the living environment. It has been demonstrated that many kinds of tumors form due to blockage of the tumor cells’ apoptosis channel, so apoptosis-inducing activities have become the common new target of antitumor drugs.

It has been confirmed that a consequent loss of mitochondrial membrane potential and an increase in reactive oxygen species are typical phenomena in the process of apoptosis related to mitochondria (4). Schwartzman and Cidlowski found that DNA fragmentation is a biological hallmark of apoptosis (5). [Ca^2+^]_i_ is an important biochemical characteristic of cell apoptosis, because increasing the [Ca^2+^]_i_ released from the intracellular Ca^2+^ pool targets the start-up of a cell's apoptosis program (6). According to the results of Reed's (7) research, another two signaling pathways, the cell death receptor pathway and the mitochondrial pathway, are involved in the mechanisms of apoptosis. In the mitochondrial pathway, the Bcl-2 family, which is well known as death signals, could trigger the release of several pro-apoptosis proteins (8). A related work showed that GFPS1b isolated from cultured mycelia of *Grifola frondosa* GF9801 was able to induce apoptosis in SGC-7901 cells accompanied with down-regulation of Bcl-2 (9). Activated caspase-3 during apoptosis is involved in the mitochondrial and cell death receptor pathways (10). *Radix hedysari* polysaccharide effectively suppresses caspase-3 overexpression in apoptosis. Emerging evidence has suggested that one of the mechanisms of the growth inhibitory effect of *Lycium barbarum* polysaccharide (LBP) on colon cancer cells is the interruption of the cell cycle. Mao 3 proved that the apoptosis-inducing activities of LBP are effected by decreasing the level of cyclin D, cyclin E, and cyclin-dependent kinase 2.

LBP is the major functional component of the fruit of *L. barbarum*, which is a well-known Chinese herb. Accumulating evidence has revealed that LBP exhibits significant antitumor activity (11–15). Various LBP samples obtained by different methods have different structures and bioactivities. However, the detailed relationship between the structures and antitumor activities of LBP samples is still unclear.

In this study, LBP was extracted from *L. barbarum* and separated by DEAE-cellulose column. The apoptosis-inducing activities of two LBP fractions (LBP-d and LBP-e) were observed with human hepatoma SMMC-7721 cells. These two LBP fractions were then purified with a Sephadex G-75 gel filtration column (Shanghai, China). Their molecular structure characteristics were investigated, and the relationship between their molecular structure and their apoptosis-inducing activities was analyzed.

## Material and methods

### Cell line

The human hepatic cancer cell line SMMC-7721 was obtained from the Cell Bank of the Shanghai Institute of Cell Biology (Shanghai, China). The cells were maintained in an RPMI 1640 cell culture medium supplemented with 10% fetal bovine serum (FBS) and antibiotics (100 U mL^−1^ penicillin and 100 µg mL^−1^ streptomycin) at 37°C in a 5% CO_2_ humidified incubator.

### Reagents

Berries from *L. barbarum* L. were purchased in Shihezi City, Xinjiang Province, China. Trifluoroacetic acid (TFA) and standard monosaccharides (D-sorbose, L-fucose, D-ribose, D-rhamnose, L-arabinose, D-galactose, D-xylose, D-mannose, and D-glucose) were purchased from Sigma-Aldrich (Shanghai, China). RPMI 1640 cell culture medium was purchased from Hyclone Laboratories, Inc. (Logan, UT, USA). FBS was purchased from Biyhjm Biosciences, Inc (Beijing, China). Acridine orange (AO), propidium iodide (PI), and 3-(4,5-dimethylthiazol-2-yl)-2,5-diphenyltetrazolium bromide (MTT) were purchased from Solarbio S&T Co. (Beijing, China). Fluo-3/AM was obtained from Beyotime Institute of Biotechnology (Haimen, China). All other chemicals and reagents were purchased locally and were of analytical grade.

### Preparation of LBP samples

Two hundred grams of dried *L. barbarum* L. berries were ground and extracted four times with 600 mL ethanol (70%) at 70°C; each extraction lasted 0.5 h. The mixture was filtrated. The residue was dried and extracted four times with 800 mL distilled water; each extraction lasted 1.5 h. The solution was vacuum-concentrated to 50 mL and precipitated by adding 80 mL of ethanol. The mixture was centrifuged (3,400 rpm, 15 min). The precipitation was dissolved in distilled water and lyophilized after protein was removed using the Sevag method. LBP was then obtained.

LBP (0.15 g) was dissolved in distilled water and applied to a DEAE-cellulose column (2.5 cm×50 cm). The column was then eluted with stepwise gradients of NaCl aqueous solutions (0, 0.05, 0.1, 0.15, and 0.2 M) at a flow rate of 2.5 mL min^−1^. The eluents of the 0.15 and 0.2 M NaCl solutions were collected and vacuum-concentrated, dialyzed for 2 days, and lyophilized. The polysaccharides obtained were named LBP-d and LBP-e, respectively; they were applied to the apoptosis-inducing experiments.

The LBP-d and LBP-e to be used for the molecular structure investigation were purified with a Sephadex G-75 gel filtration column (φ 25 mm×400 mm). The flow rate was 28 mL h^−1^. The eluents were vacuum-concentrated, dialyzed, and freeze-dried.

### Composition and structure analysis

#### Uronic acid, protein, and neutral sugar determination

The uronic acid content of the purified fractions was determined by the sulfuric acid carbazole assay (16), and galacturonic acid was used to make a standard curve. Absorbance of the samples was measured at 525 nm. The protein content was determined according to the method of Bradford (17), standardized using bovine serum albumin. The neutral sugar content of LBP was analyzed by the modified phenol-sulfuric method standardized using D-glucose (18).

### Monosaccharide component analysis

LBP-d and LBP-e were treated with 2 mol L^−1^ trifluoroacetic acid (TFA) at 120°C for 6 h; after that, TFA was eliminated by vacuum rotary evaporation to dryness. The hydrolyzed polysaccharide sample, rhamnose (Rha), fucose (Fuc), arabinose (Ara), xylose (Xyl), mannose (Man), galactose (Gal), glucose (Glu), sorbose (Sor), and ribose (Rib) as monosaccharide standard were dissolved in distilled water (2 mL), respectively. The solution was then reduced by sodium borohydride (NaBH_4_, 30 mg) for 1.5 h, treated with glacial acetic acid (AcOH) to decompose excessive NaBH_4_, and dried by rotary evaporation under reduced pressure at 60°C. Subsequently, 2 mL 0.1% (v/v) HC1-methanol was added, and the solution was washed with a small amount of distilled water by oscillation (repeating four times). The remaining mixture was dried at 105°C, added to 0.5 mL pyridine and 0.5 mL acetic anhydride, then heated to complete the reaction at 100°C for 1 h. The mixture was then subjected to GC analysis.

GC conditions were as follows: OV1701 silica capillary column (Tokyo, Japan), 30 m×0.32 mm×0.5 µm; initial column temperature of 150°C, which was increased to 240°C at a rate of 10°C min^−1^; inlet temperature of 250°C; helium flow rate of 1 mL min^−1^.

### Periodate oxidation and Smith degradation

The standard curve was drawn for sodium iodate consumption. Solutions of NaIO_4_ (0.015 mol L^−1^, 50.0 mL) and NaIO_3_ (0.015 mol L^−1^, 50.0 mL) were prepared. They were then combined in proportions of 5–0, 4–1, 3–2, 2–3, 1–4, and 0–5. The mixture (0.4 mL) was diluted to 100 mL and the absorption at 223 nm was measured to get the standard curve. The LBP (20 mg) was dissolved with 30 mL of 0.015 mol L^−1^ NaIO_4_. The mixture was kept in the dark at 4°C until the optical density at 223 nm did not increase any longer. The consumption of NaIO_4_ was determined by the standard curve and formic acid production was determined by titration with sodium hydroxide (0.01 mol L^−1^).

The reaction mixture was reduced by sodium borohydride (NaBH_4_), neutralized by 0.1 mol L^−1^ acetic acid, dialyzed against distilled water, and the retentate was then lyophilized to yield the polyalcohol. The polyalcohol was treated with TFA (2 mol L mol L^−1^, 2 mL) at 120°C for 2 h, dissolved in distilled water (2 mL), reduced by sodium borohydride (NaBH_4_, 30 mg) for 1.5 h, treated with glacial acetic acid (AcOH) to decompose excessive NaBH_4_, and vacuum-dried at 60°C. The residues were then mixed with 2 mL 0.1% (v/v) HC1-methanol. The mixture was washed four times with a small amount of distilled water and was dried at 105°C, followed by the addition of 0.5 mL pyrimidine and 0.5 mL acetic anhydride. The mixture was heated to 100°C for 1 h and was subjected to GC analysis.

### MTT colorimetric assay

The inhibition effects of LBP on SMMC-7721 cells were measured by MTT assay. Briefly, SMMC-7721 cells in exponential growth were seeded at a final density of 5×10^5^ cells mL^−1^ in a 96-well plate. After incubation for 24 h, the cells were treated with LBP (0, 50, 100, 200, and 400 mg L^−1^) for 2, 4, and 6 days. The medium was replaced every other day. After cultivation, 40 µl MTT (1 mg mL^−1^) was added and the cells were further cultured for 4 h, then the supernatant was discarded and 100 µl DMSO was added. The absorbance of each well was measured at 490 nm on an ELISA reader. The percentage of cell viability was calculated as a ratio of the OD value of the sample to the OD value of the control.

### Morphological observation

SMMC-7721 cells (2×10^4^) were cultured with a basal medium and a medium containing 400 mg L^−1^ LBP-d or LBP-e, respectively, for 4 days. The morphological observation was then conducted with an inverted microscope and laser scanning confocal microscope (LSCM) (Nicon, Japan). The cells were dyed by AO before being examined by LSCM.

#### Analysis of cell-cycle phase distribution by flow cytometry with PI staining

Cell cycle analysis was performed using a hypotonic solution of PI. SMMC-7721 cells were treated with 400 mg L^−1^ LBP-d or LBP-e for 4 days. Approximately 8×105 cells were harvested, washed twice with cold PBS, and fixed with ice-cold 70% ethanol for at least 24 h. The fixed cells were centrifuged at 1,500 r min^−1^ for 5 min and resuspended in PBS. The cell suspension was stained using PI (50 µg mL^−1^; Sigma-Aldrich) and RNase A (100 µg mL^−1^; Sigma-Aldrich) in the dark at 37°C for 30 min and then analyzed by flow cytometry.

### Determination of the concentration of calcium in cells

SMMC-7721 cells (2×10^4^) were cultured for 4 and 6 days in a medium containing 400 mg L^−1^ LBP-d or LBP-e, respectively. Then the fluo-3/AM (Sigma-Aldrich) was added to cells for an additional 40-min incubation. Finally, the cells were washed three times with PBS and examined by LSCM.

### Statistical analysis

The results are presented as means±SD. Significant differences were evaluated with a Student's t test.

## Results and discussion

### Compositions of LBP

Some polysaccharides contain neutral sugar and uronic acid, which usually conjugates with protein and is closely related to the bioactivities of polysaccharides. Thus it is necessary to analyze the contents of neutral sugar, uronic acid, and protein in LBP. As shown in Table 1, the neutral sugar content of LBP-d was higher (71.2%) than that of LBP-e (67.3%), but the uronic acid and protein contents of LBP-d were lower (16.0%, 2.1%) than those of LBP-e (23.2%, 3.1%). According to gas chromatography analysis, LBP-d was composed of eight kinds of monosaccharides: fucose, ribose, rhamnose, arabinose, xylose, mannose, galactose, and glucose, with molar ratios of 19.6, 1.5, 28.9, 6.3, 1.6, 6.2, 21.5, and 14.3. LBP-e was composed of six kinds of monosaccharides: fucose, rhamnose, arabinose, mannose, galactose and glucose, with molar ratios of 5.5, 8.8, 1.7, 35.2, 3.4, and 45.4 (Table 1). The above results differed from previous reports, which concluded that LBP consisted of nine kinds of monosaccharides (19). Polysaccharide samples are a mixture of many kinds of polysaccharide fractions with different compositions. Extraction and purification methods could alter the polysaccharide molecules, thus changing the monosaccharide composition.

**Table 1:** Results of composition analysis and periodate oxidation of LBP-d and LBP-e

Sample	LBP-d	LBP-e
Protein (%)^a^	2.1±0.2	3.1±0.1
Neutral sugars (%)^a^	71.2±2.3	67.3±2.8
Uronic acid (%)^a^	16.0±0.3	23.2±0.4
Consumption of periodate oxidation (µmol)^a^	75.4	56.8
Generation of formic acid (µmol)^a^	12.1	8.1
Sugar composition (mol%)^a^		
Fucose	19.6	5.5
Ribose	1.5	Not detected
Rhamnose	28.9	8.8
Arabinose	6.3	1.7
Xylose	1.6	Not detected
Glucose	6.2	35.2
Mannose	21.5	3.4
Galactose	14.3	45.4

a Data are shown as mean±standard deviation, *n*=3. LBP, *Lycium barbarum* polysaccharide.

### Periodate oxidation and Smith degradation

**Fig. 1:**
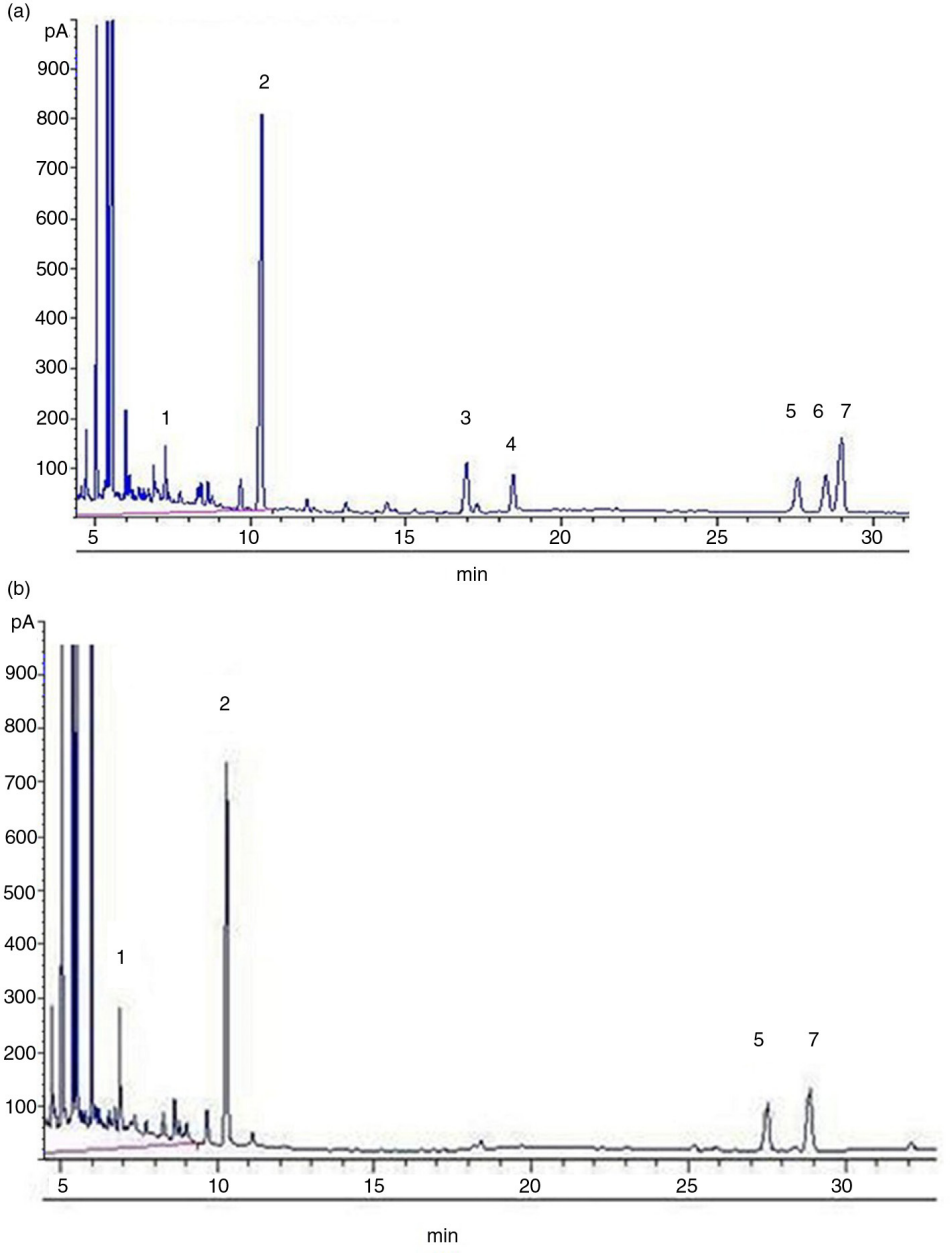
Gas chromatographs of Smith degradation of LBP-d and LBP-e. a: LBP-d, b: LBP-e. 1: Glycerol, 2: erythritol, 3: fucose, 4: arabinose, 5: glucose, 6: mannose, 7: galactose.

The results of periodate oxidation and Smith degradation were shown in Table 1 and Fig. 1. LBP-d and LBP-e were each oxidized with 0.015 mol L^−1^ sodium metaperiodate (NaIO_4_) at 4°C in the dark until the reaction was completed. During the reaction, 75.4 and 56.8 µmol of NaIO_4_ was consumed, and 12.1 and 8.1 µmol of formic acid was liberated per mole of sugar residue for LBP-d and LBP-e, respectively. Those results indicated that the molar ratios (%) of glycosyl with two joint hydroxyls to glycosyl with three joint hydroxyls and other residues were 11.2:47.4:41.4 and 7.4:37.3:55.3 in LBP-d and LBP-e, respectively. We thus deduced the existence of 1→6, 1→2, 1→4, and 1→3 linked hexapyranose residues and 1→5, 1→2, and 1→3 linked furanose residues in LBP-d and LBP-e. The Smith degradation resulted in a large amount of glycerol and erythritol, indicating that there were 1→, 1→2, 1→6, 1→2,6, 1→4, and 1→4,6 linkages in LBP-d and LBP-e. The presence of galactose, fucose, glucose, and mannose indicated the existence of (1→3)-linked, (1→2,3)-linked, (1→2,4)-linked, and (1→3,4)-linked glucose, fucose, and mannose residues. The presence of arabinose revealed that some arabinose residues were (1→3)-linked or (1→2)-linked.

### Growth inhibitory effects of LBP on SMMC-7721

The results of the MTT assay are summarized in Table 2. LBP exhibited a significantly suppressive effect on the growth of SMMC-7721 cells. The maximum inhibition ratios of LBP-d and LBP-e were 26.70% (*P*<0.01) and 45.13% (*P*<0.01) at a dose of 400 mg/L for 4 days (Table 2). Thus the subsequent experiments were also conducted with a concentration of 400 mg L^−1^.

**Table 2:** Inhibition activities of LBP-d and LBP-e on SMMC-7721 cells

	Time (d)	Concentration (mg L^−1^)	*n*	Inhibition ratio (%)
		400	10	23.83±8.90^Aa^
	2	200	10	12.40±9.36^Bb^
		100	10	9.06±7.08^Bb^
		50	10	7.50±6.97^Bb^
		400	10	26.70±8.90^Aa^
LBP-d	4	200	10	10.50±7.77^ABb^
		100	10	8.86±7.39^Bb^
		50	10	19.71±7.16^ABab^
		400	10	10.10±9.22^a^
	6	200	10	16.46±9.49^a^
		100	10	14.21±8.05^a^
		50	10	15.61±6.00^a^
		400	10	17.13±12.33^a^
	2	200	10	16.29±13.29^a^
		100	10	18.21±13.96^a^
		50	10	12.03±9.30^a^
		400	10	45.13±5.69^A^
LBP-e	4	200	10	23.52±13.47^B^
		100	10	17.91±8.75^B^
		50	10	15.01±9.37^B^
		400	10	37.07±10.31^a^
	6	200	10	29.46±9.13^a^
		100	10	33.28±8.14^a^
		50	10	32.06±9.51^a^

Letters show significant differences. A, B: *P*<0.1. a, b: *P*<0.05.

The finding that LBP had an inhibitory effect on the growth of hepatoma cells was consistent with a previous report (20). Zhang et al. reported that MAP, a novel polysaccharide from the loach (*Misgurnus anguillicaudatus*), inhibited SMMC-7721 cell growth in a time- and concentration-dependent manner (6). However, this finding was slightly different from our results, which may be due to the different structural characteristics and action modes of polysaccharide.

### Morphological observation

**Fig. 2:**
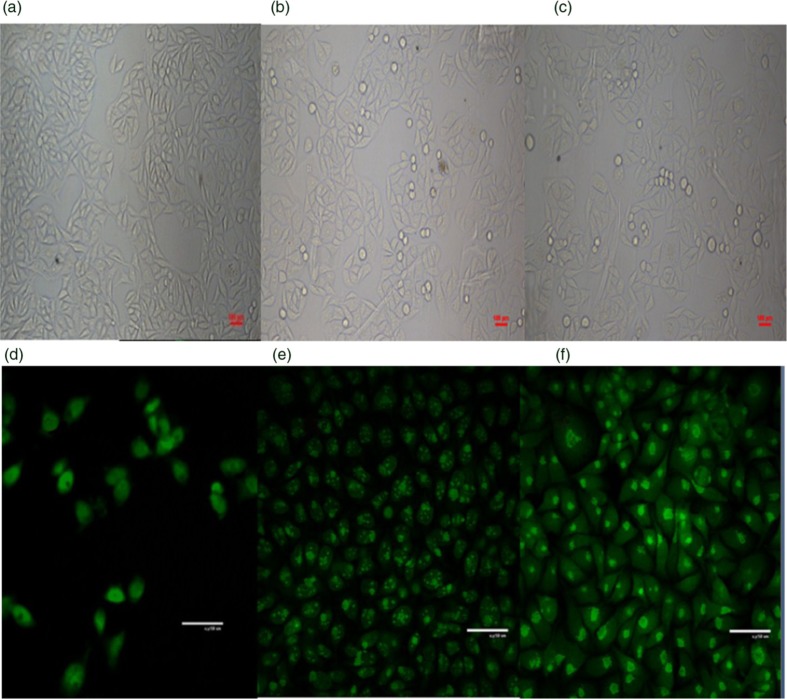
Morphological changes of cells treated by LBP. a, b, c show images of SMMC-7721 cells observed by inverted microscope (500×), and d, e, f are images observed by laser scanning confocal microscope (500×); a and d are the control groups; b and e are cells treated with LBP-d (400 mg L^−1^) for 4 days; c and f are cells treated with LBP-e at 400 mg L^−1^ for 4 days.

The effects of LBP on the morphological characteristics of SMMC-7721 cells are shown in Fig. 2. Figure 2a shows images of control group cells observed by inverted microscope. The cells are closely spaced with a uniform arrangement, distinct cell borders, and a strong refraction rate. They appear triangular and spindly in shape and are of consistent size. Images of cells treated with LBP-d and LBP-e observed by inverted microscope are shown in Fig. 2b and 2c. The treated cells shrank, elongated, or became round with a decreasing of density. Some cells became suspended in the medium. The results of our research indicated that when apoptosis occurred, the cells revealed marked shrinkage, condensation, and detachment from the culture plate (21, 22). The characteristics of LBP-treated cells proved that LBP was able to induce apoptosis in SMMC-7721 cells. Images of SMMC-7721 cells observed by LSCM (Fig. 2e and 2f) show that the nuclei of cells treated by LBP-d broke into fragments, but that of cells treated by LBP-e shrank and moved to the edge of the cells. In apoptosis, morphological changes can be observed, including chromatin condensation, nuclei pycnosis, DNA fragmentation, and apoptotic body formation (23). These phenomena were similar to those of our experimental results, confirming the apoptosis-inducing activity of LBP on SMMC-7721 cells. Meanwhile, differences in the morphological characteristics of cells treated with LBP-d and LBP-e suggest that these two kinds of LBP fractions have various mechanisms of apoptosis-inducing activity.

### Effects of LBP on the cell cycle of SMMC-7721 cells

To prove whether LBP-induced apoptosis was related to arrest of SMMC-7721 cell cycle progression, FCM was used to quantify the induction of apoptosis and the effect on cell cycle distribution by LBP. The effects of LBP on the cell proliferation cycle are shown in Table 3. LBP-d increased the percentage of G0/G1 phase cells from 74.6 to 85.9% and decreased the percentage of S phase cells from 24.8 to 13.4%, suggesting that it blocks the proliferation of SMMC-7721 cells in the G0/G1 phase. On the contrary, LBP-e increased the percentage of S phase cells from 24.8 to 45.4% and decreased the percentage of G0/G1 phase cells from 74.6 to 51.0%, indicating that it blocked the proliferation of SMMC-7721 cells in the S phase.

**Table 3:** Effects of LBP-d and LBP-e on cell cycle distribution of SMMC-7721 cells (*n*=3)

Group	G0/G1(%)	S(%)	G2/M(%)
Control	74.6±0.8	24.8±1.4	0.6±0.2
LBP-d^a^	85.9±0.9**	13.4±0.8**	0.7±0.3
LBP-e^a^	51.0±1.6**	45.4±1.2**	3.6±0.9*

a The concentrations of LBP-d and LBP-e were all 400 mg L^−1^. All cells were cultured for 4 days.

* *P*<0.05

** *P*<0.01, compared with the control group.

The tumor cells had their own growth cycle. In general, the G0/G1 phase lasted the longest, and the ratio of cells in this phase was the highest. Compared with the G0/G1 phase, the G2/M phase period was short, so the ratio of cells was lowest in the G2/M period. Different antitumor drugs had selectivity on different proliferation cycles. For example, Gekko sulfated polysaccharide–protein complex was able to block SMMC-7721 cells in the S phase (24), while the combination of β-aescin and 5-fluorouacil agents arrested these cells in the G0/G1 phase (25). Hu et al. (1) found that different doses of FAC arrested SMMC-7721 cells in the G0/G1 and S phases, to different degrees. Our results indicated that different LBP fractions had various effects on cell cycle, the detailed mechanisms of which are still unknown and require further study.

### Effects of LBP on intracellular calcium

The effects of LBP-d and LBP-e on the concentration of Ca^2+^ in the cytoplasm of SMMC-7721 cells are shown in Table 4. Both LBP-d and LBP-e enhanced the concentration of Ca^2+^ in the cytoplasm of SMMC-7721 cells, which was 6.60 and 6.57 times greater than that of the control group, respectively (Table 4). However, when the time of treatment reached 6 days, the [Ca^2+^]_i_ of the LBP-d and LBP-e treated groups was similar to that of the control group.

**Table 4:** Effects of LBP-d and LBP-e on concentration of Ca^2+^ in the cytoplasm of SMMC-7721 cells

Group	Time (days)	*n*	Fluorescence intensity of control group (×10^3^)	Fluorescence intensity of treated group (×10^3^)	Treated/control
LBP-d	4	10	5.24±1.87	34.60±18.34**	6.60
	6	10	5.24±1.87	7.79±1.06*	1.49
LBP-e	4	10	1.94±0.51	12.74±2.02**	6.57
	6	10	1.94±0.51	2.25±1.06	1.16

The concentration of calcium was expressed in the relative fluorescence intensity of cells.

* *P*<0.05

** *P*<0.01, compared with the control group.

Considerable experimental evidence has suggested that calcium ions are associated with cell apoptosis. Some research has proven that calcium ions released from the endoplasmic reticulum into the cytoplasm are absorbed by the mitochondria. This release causes an overload in the concentration of calcium ions in the mitochondria, which leads to mitochondrial injury, cytochrome C release, and the activation of caspases and finally induces cell apoptosis (26, 27). The increase of intracellular-free [Ca^2+^]_i_ included two basic pathways. One was concerned with intracellular calcium stores; another was relative to extracellular calcium ion influx (28–32). When cells were stimulated, the Ca^2+^ pool in the endoplasmic reticulum released calcium ions, increasing the concentration of Ca^2+^ cytoplasm, which was targeted as a signal to start apoptosis (33–35). Our results proved that LBP was able to induce apoptosis by enhancing [Ca^2+^]_i_ in hepatoma cells. This finding was in agreement with a previous study (2).

The composition and structure of polysaccharides were strongly related to their antitumor activities (36). Reports showed that the contents of protein, galactose, and uronic acid in polysaccharides played an important role in their bioactivities (37–39). According to the above data, we inferred the following conclusion: One of the factors influencing the advanced polysaccharide structure was hydrogen bonding, which could be affected by uronic acid. Different uronic acid and sugar composition between LBP-e and LBP-d induced different advanced structures of polysaccharides, and they displayed different activity, illustrating that bioactivity was decided by the advanced structure in polysaccharide. A possible mechanism of apoptosis was that LBP induced some factors in SMMC-7721 cells, which mediated a release of Ca^2+^ from the intracellular Ca^2+^ pool, and increased [Ca^2+^]_i_ triggered the cells to start up the apoptosis program. Because of the different advanced structures between LBP-d and LBP-e, for example forming a cobweb, chains, or a ball, active materials might be wrapped or exposed in LBP-d and LBP-e, resulting in different active effects.

Our results showed that the contents of protein, uronic acid, and galactose in LBP-e were much higher than those in LBP-d, which might responsible for the bioactivity differences between LBP-e and LBP-d.

## Conclusion

Two LBP fractions (LBP-d, LBP-e) with apoptosis-inducing activities were obtained. They were composed of protein, neutral sugars, and uronic acid in different proportions. LBP-d blocked SMMC-7721 cells at the G0/G1 phase with an inhibition ratio of 26.70%, while LBP-e arrested SMMC-7721 cells at the S phase with an inhibition ratio of 45.13%. Both LBP fractions induced apoptosis by enhancing the concentration of Ca^2+^ in the cytoplasm of SMMC-7721 cells. The protein, uronic acid, and galactose contents in LBP-e were much higher than those in LBP-d, which might responsible for the bioactivity differences between LBP-e and LBP-d.
